# Endoscopic negative pressure therapy as a salvage treatment for management of post-surgical anastomotic leaks without ostomy after colorectal resection

**DOI:** 10.1038/s41598-025-25181-3

**Published:** 2025-10-31

**Authors:** Marcus Kantowski, Daniel Perez, Eugen Bellon, Thomas Rösch, Ali Ramouz, Michael Tachezy, Pasquale Scognamiglio

**Affiliations:** 1 Department of Interdisciplinary Endoscopy, University Medical Center Hamburg-Eppendorf, 20248 Hamburg, Germany; 2 Department of General, Visceral and Thoracic Surgery, University Medical Center Hamburg-Eppendorf, 20248 Hamburg, Germany; 3 Department of Surgery, Heidelberg University Hospital, 69120 Heidelberg, Germany; 4 Department of General, Visceral and Thoracic Surgery, University Hospital of Heidelberg, Im Neuenheimer Feld 420, 69120 Heidelberg, Germany

**Keywords:** Leakage, Complication management, Vacuum therapy, Surgery, Rectal cancer, Anatomy, Diseases, Signs and symptoms

## Abstract

Anastomotic leakage (AL) after lower gastrointestinal surgery remains a serious complication with an incidence of 3–30%. Influencing factors include perfusion deficits, tension, inflammation, malnutrition, medications, and previous therapies. Surgical technique and type of anastomosis are also crucial. While various strategies for early detection and management exist, endoscopic negative pressure therapy (ENPT) has shown promising therapeutic benefits. This study evaluates the technique and outcomes of ENPT for treating AL. In this retrospective analysis, 59 patients with grade B AL following rectal resection between June 2016 and February 2022 were included. Patients had endoscopically or radiologically confirmed AL with pelvic abscess and were treated with ENPT. Exclusion criteria included relaparotomy, CT-guided drainage, sealant use, and irrigation without ENPT. Outcomes included leakage closure, abscess recurrence, sepsis, ostomy closure rate, and complications. Of the 59 patients, 16 received ENPT without an ostomy; 10 of these achieved complete healing. Median ENPT duration was 4 weeks. Higher anastomosis levels correlated with better healing. Factors such as leak type, abscess size, and chronicity influenced outcomes. Reported complications included rectovaginal fistula and anastomotic stenosis. ENPT is a safe and effective approach for AL, often avoiding permanent ostomies. A structured, endoscopy-led treatment strategy is essential.

**Supplementary Information:**

The online version contains supplementary material available at 10.1038/s41598-025-25181-3.

## Introduction

Anastomotic leakage (AL) following lower gastrointestinal tract surgery is a serious concern, with incidence rates varying from 3% to 30%, dependent on the type of surgery and the location of the anastomosis^1,2^. Factors contributing to AL include circulatory issues, suboptimal suture tension, inflammation, malnutrition, and certain medications such as cortisone or those used in chemotherapy, as well as previous radiotherapy or chemotherapy^3^. Notably, a surgeon’s technical proficiency and the anastomosis method employed are crucial factors affecting the occurrence of AL. Most cases of present between postoperative days 4 and 10; however, clinically significant AL may not manifest until several weeks or even months after surgery, especially following radiotherapy or chemotherapy^4^. There is debate over the necessity and timing of routine examinations to detect early AL during a normal postoperative course^5^. The possible early symptoms of AL include fever, cardiorespiratory difficulties, diarrhea, acute renal failure indicative of emerging sepsis, elevated infection markers in the blood, and otherwise unexplained accumulations in local drains^6^.

In the event of postoperative complications or raised inflammatory markers, an anastomosis review is mandatory^7^. This can involve direct endoscopic examination or radiological assessment. In facilities with advanced endoscopic capabilities, routine evaluations of the anastomosis are performed endoscopically, offering substantial safety benefits. Endoscopy can detect minor AL and circulatory problems that may be missed radiologically^8^. After a long period in which surgical revision was favored, the balance has now swung towards non-surgical, predominantly endoscopic forms of intervention. These include a wide array of treatment options depending on the AL size and the presence of pelvic abscesses: injection methods, mechanical closure with various clips, stenting, and local drainage—either passive via an endoscopically placed tube or active with endoscopic negative pressure therapy (ENPT). The latter is now considered the standard treatment in many colorectal centers, with published healing rates of around 80% and few treatment-related adverse events^9–12^.

In instances of late AL diagnosis or critical conditions such as severe sepsis, organ failure, or peritonitis, swift surgical intervention is imperative. The options include lavage, Hartmann’s procedure, redo anastomosis surgery, and a protective ostomy. However, these surgical procedures can significantly distress patients and are associated with morbidities, particularly in the elderly and those with comorbidities, who may suffer long-term consequences from a permanent ostomy. Therefore, in stable patients, management of leakage with ENPT alone is a rational approach to reduce ostomy rates. Meta-analyses show that ostomy closure rates after ENPT for rectal AL vary widely, from 31% to 100%. Early ENPT without ostomy creation has shown promising results in small subgroups^13,14^.

This study aims to present the authors’ experience with their method for the treatment of patients with AL, focusing on factors influencing the success of endoscopic therapy. Generally, the postoperative period of stabilization and effective AL healing is expected to last 2–4 weeks, although this can be extended in patients with prior radiotherapy^15^. In such cases, endoscopic therapy without ostomy has been attempted. This article presents standardized ENPT as well as new techniques and approaches for dealing with challenges such as abdominal cavity access or sponge occlusion by fecal contamination.

## Results

**Fig. 1:**
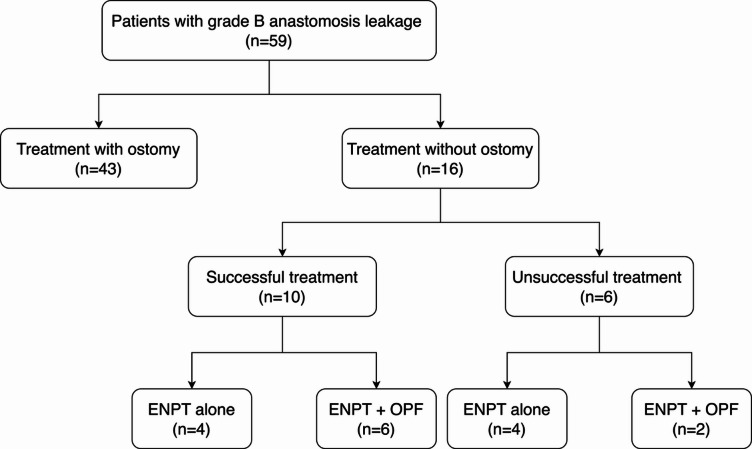
Flowchart of the patients included in the study based on the treatment success.

Overall, 59 patients with grade B AL after rectal resection were included in our retrospective analysis, conducted from June 2016 to February 2022 at the University Medical Center Hamburg-Eppendorf, Germany. Endoscopic treatment took the form of ENPT and protective ostomy in most cases. In 16 patients, an ostomy was technically infeasible due to the local anatomy (carcinomatosis, adhesions, or obesity) or because the patients refused the ostomy operation (Fig. 1). These patients’ demographic and clinical characteristics are listed in Table 1. The indication for primary operation was colorectal cancer in seven of these patients. Of the remaining patients, five had diverticular disease, three underwent colorectal resection as part of an operation for other local malignancies, and one had chronic inflammatory disease. As for reconstruction, in ten cases a descendo-rectostomy was performed, three patients received a coloanal anastomosis, and the three remaining patients had already received an intestinal anastomosis. Seven of the 10 patients with oncological disease underwent neoadjuvant radiotherapy or chemotherapy before surgery.

**Table 1:** Demographic and peri-interventional characteristics of patients with grade B anastomotic leakage.

		Successful treatment	Unsuccessful treatment	*p*-value
		(*n* = 35)	(*n* = 24)	
Age		60 (35–70)	60 (18–78)	0.739
Body-mass index (BMI)		23.8 (16.8–32.4)	25.3 (18.9–36.7)	0.052
Gender	Male	22 (62.9%)	16 (66.7%)	0.79
	Female	13 (37.1%)	8 (33.3%)	
Indication	CRC	22 (64.7%)	12 (50.0%)	0.483
	Other malignancies	5 (14.7%)	5 (20.8%)	
	Diverticulosis	5 (14.7%)	3 (12.5%)	
	IBD	2 (5.9%)	4 (16.7%)	
Neoadjuvant treatment	Chemotherapy	3 (27.3%)	5 (41.7%)	0.667
	Total neoadjuvant therapy (TNT)	8 (72.7%)	7 /58.3%)	
Anastomosis type	Ileocolic	7 (20.0%)	5 (20.0%)	0.401
	Colorectal	19 (54.3%)	17 (68.0%)	
	Coloanal	9 (25.7%)	3 (12.0%)	
Anastomosis configuration	Side-to-side	12 (34.3%)	8 (33.3%)	1.000
	End-to-side	23 (65.7%)	16 (66.7%)	
Anastomosis technique	Hand-sewn	9 (25.7%)	8 (33.3%)	0.569
	Stapler	26 /74.3%)	16 (66.7%)	
Anastomotic insufficiency	Leakage	30 (88.2%)	11 (45.8%)	0.002
	Abscess	2 (5.9%)	8 (33.3%)	
	Fistula	2 (5.9%)	5 (20.9%)	

All patients were clinically stable with relative contraindications for treatment of the AL without ostomy by ENPT, including complete ostomy refusal (*n* = 12), severe adhesions in the index operation (*n* = 11), an earlier unsuccessful attempt at AL revision (*n* = 6), severe comorbidities (*n* = 3), peritoneal carcinomatosis (*n* = 2), and obesity (*n* = 1). Prerequisites for this concept were cooperation by the patient and a manageable estimated duration of therapy on initial endoscopy of approximately 4 weeks. ENPT was implemented for the treatment of 10 patients (62.5%) with acute AL and six patients (37.5%) with chronic AL. Three patients had an abscess in the pelvic cavity due to the AL. AL with a communication towards the free abdominal cavity was diagnosed in four patients. In these cases, food or feces were removed from the abdominal cavity and lavage was performed under endoscopic guidance. AL was treated by endoscopic cleanout/irrigation of the abscess cavity or free abdominal cavity, intracavitary vacuum therapy, and sealing to the lumen. In these four cases, the abdominal cavity could be better reached percutaneously using a pre-existing drainage channel, so that lavage of the abdominal cavity following ENPT with white sponge or OPF was performed in this way. In eight patients, OPF was used after the black sponge in the later healing process to reduce the size of the abscess. With preserved fecal passage, the vacuum sponge in the abscess quickly becomes obstructed, so the intestinal lumen was rectally irrigated several times a day during treatment as needed, by means of enema, rectal irrigator, decompression probe, or irrigation system. In four patients, larger suture defects were sealed using endoscopically placed Vicryl mesh (Ethicon), which was changed endoscopically every 3–6 days until no longer needed.

Successful treatment was defined as complete closure of the anastomotic defect and good functional outcome (Table 2). Complete healing of AL without ostomy was achieved in 10/16 patients. The median duration of ENPT treatment was 4 weeks (range 1–8). Patients receiving a higher colorectal anastomosis had a better healing rate than those with an anastomosis close to the anal verge. The kind of leakage also affected treatment success, isolated AL being more likely to heal than a pelvic abscess or a fistula to other organs. The size of the abscess had no statistically significant association with treatment success.

**Table 2:** Demographic and peri-interventional characteristics of the patients with grade B anastomotic leakage who underwent endoscopic treatment.

		Successful treatment	Unsuccessful treatment	*p*-value
		(*n* = 10)	(*n* = 6)	
Age		54 (41–89)	59 (28–84)	0.482
Body-mass index (BMI)		25.1 (19.8–32.4)	25.9 (21.0-36.7)	0.172
Gender	Male	3 (30%)	3 (50.0%)	0.607
	Female	7 (70%)	3 (50.0%)	
Indication	CRC	4 (40.0%)	3 (50.0%)	0.483
	Other malignancies	2 (20.0%)	1 (16.7%)	
	Diverticulosis	4 (40.0%)	1 (16.7%)	
	IBD	0 (0.0%)	1 (16.7%)	
Neoadjuvant treatment	Chemotherapy	3 (30.0%)	1 (16.7%)	0.486
	Total neoadjuvant therapy (TNT)	1 (70.0%)	2 (33.3%)	
Anastomosis type	Ileocolic	2 (20.0%)	1 (16.7%)	0.965
	Colorectal	6 (60.0%)	4 (66.6%)	
	Coloanal	2 (20.0%)	1 (16.7%)	
Anastomosis technique	Hand-sewn	2 (20.0%)	2 (33.3%)	0.604
	Stapler	8 (80.0%)	4 (66.7%)	
Type of anastomosis leakage	Acute	3 (30.0%)	3 (50.0%)	0.607
	Chronic	7 (70.0%)	3 (50.0%)	
Anastomosis configuration	Side-to-side	2 (20.0%)	1 (16.7%)	1.000
	End-to-side	8 (80.0)	5 (83.3%)	
Time of leakage (days after surgery)		33 (19–90)	25 (7–38)	0.181
Number of endoscopy sessions		7 (2–15)	7 (2–14)	0.792
Duration of anastomosis leakage (month)		1 (1-192)	1 (1–84)	0.987
Duration of endoscopic therapy (weeks)		3 (1–6)	4.5 (1–8)	0.147
Conversion to open pore film-drainage (OPF)		6 (60.0%)	2 (33.3%)	0.580
Reoperation		2 (20.0%)	2 (33.3%)	0.614

No significant differences in terms of treatment success were found for the indication for surgery, neoadjuvant radiation, type of operation, reoperation during endoscopic treatment, treatment duration, or time from leakage diagnosis to start of ENPT. Neither the different techniques and materials of ENPT nor the additional endoscopic methods showed any differences in terms of complications. The endoscopic treatment was started at a median of 33 days after surgery (range 7–90) and took a median 30 days. A median of six endoscopies per patient were performed (range 2–15).

One patient with a chronic AL stopped the ENPT after two sessions in 1 week without healing of the defect. In an 84-year-old patient with AL after sigmoid resection who developed a rectovaginal fistula during ENPT, treatment was stopped immediately and a transversostomy was carried out. Four patients showed no relevant tendency towards healing of their AL after 4 weeks of ENPT. Two of these patients had long-term AL (18 and 192 months), while in the other two the AL had been present for 2 and 3 months respectively. In three of these patients a second operation with ostomy was performed. One patient with minor leakage from a hand-sewn coloanal anastomosis (after radiochemotherapy with anastomosis) developed symptoms of low anterior resection syndrome (LARS). She declined a second operation and her rectal functional problems persisted (high LARS score with symptoms of urge and diarrhea).

### Treatment-related complications

Complications occurred in three patients (19%). In an 84-year-old patient with AL after sigmoid resection who developed a rectovaginal fistula during ENPT, treatment was stopped immediately and a transversostomy was carried out. One patient developed stenosis of the anastomosis as a complication of leakage months after the end of ENPT, necessitating two further sessions of endoscopic treatment with balloon dilatation. The third patient developed a symptomatic anastomotic stenosis after three years. An endoscopic incision and balloon dilatation were needed to restore acceptable anastomotic diameter.

## Discussion

The mortality rate following colorectal surgery is approximately 7.5%, with AL occurring in most of these cases (6.6%). Sepsis (30%), prolonged ventilation (43%), peritonitis (26%), and failure to rescue (25%) are leading causes of mortality^16,17^. Given that 90% of patients who undergo rectal resection do not develop AL, routine protective ostomy may not be necessary for all patients. However, in cases where AL does occur, maintaining a “clean” anastomotic region to prevent pelvic or intra-abdominal contamination is crucial. Protective ostomy facilitates further treatment by keeping the anastomotic region clear of contaminants, thereby improving morbidity, mortality, length of hospital stay, the oncological outcome, and long-term survival^17^. To the best of the authors’ knowledge, this is one of the few studies comprehensively evaluating the effectiveness of ENPT for treating AL after colorectal surgery. While the benefits of protective ostomy in reducing the incidence and severity of AL are well documented, there remains a need for advanced treatments such as ENPT for patients who develop AL despite prophylactic measures.

ENPT has become an established therapy for anastomotic leakage after colorectal surgery, boasting a success rate of 80%^18^. Our study reports an overall successful closure rate of 63% of patients achieving complete closure solely through ENPT within this period, among patients who could not receive fecal diversion mainly due to medical and surgical issues. This success rate underscores ENPT’s efficacy as salvage option for AL in the absence of fecal diversion, particularly in managing small to medium-sized leaks. However, for patients with total AL or segmental necrosis, reoperation is often necessary to re-establish intestinal continuity due to the high risks of poor functional outcomes, fistula formation, or late local abscesses, even under optimal ENPT conditions.

The selection of patients is critical for the success of ENPT. Our data indicate that patients receiving ileo-colorectal anastomoses with protective ostomies had better healing outcomes than those with terminal ostomies. Because the probable reason for this is that terminal ostomies are often performed in patients with peritonitis or significant comorbidities, such as immunosuppression. Simple anastomotic leaks showed a higher rate of healing than pelvic abscesses or fistulas involving other organs. Chronic fistulas presented more challenges, requiring longer treatment durations and more interventions than acute fistulas, although the differences were not statistically significant. Despite the proven efficacy of ENPT, complications such as recurrent abscess or fistula can occur following treatment^18^. This necessitates ongoing refinements in ENPT protocols and individualized treatment plans. Innovations such as the VAC stent for gastrointestinal leaks, which combines a covered metal stent with sponge segments, offer promising alternative approaches^19^. Additionally, our use of transanal wound rinsing as an additive treatment during ENPT (ENPT/TRT) has been shown to improve outcomes and facilitate outpatient care^20^.

Moreover, it is essential for practitioners to learn and master ENPT and its potential pitfalls without harming critically ill patients^21^. The necessity of individualized treatment is becoming more evident, with modifications of the standard ENPT constantly being described in the literature. Factors exerting a significantly negative influence on the success of ENPT include multivisceral resections, recent surgical revisions, and extended duration of treatment, as highlighted by Kühn et al. in their study of 281 patients^11^.

Our study suggests that a structured approach, supported by a dedicated endoscopic team, is crucial for optimizing patient outcomes. Early intervention with ENPT significantly improves clinical outcomes, as confirmed by meta-analyses^22^. This aligns with our findings, where the closure rate was higher in patients who received early treatment than in those with chronic fistulas. In our cohort, the median duration of endoscopic treatment was 4 weeks, with a median of four endoscopic procedures per patient, fewer than the reported average of seven procedures in other studies. Repeated endoscopic interventions allow for effective lavage and debridement, reducing perianastomotic inflammation. A significant number of patients were treated on an outpatient basis, which underscores the capacity of ENPT to reduce the length of hospital stay and the associated healthcare costs. The retrospective nature and limited sample size of our study restrict the generalizability of our results. Nonetheless, our findings provide valuable insights into the factors influencing the success of ENPT. Complications in our cohort were consistent with literature reports, with anastomotic stenosis being the most frequent. Potential treatment-related complications include bleeding, persistent fistula, and abscess/sinus formation, although these were not observed in our cohort. Based on our experience, the risk of bleeding due to sponge ingrowth can be mitigated by shorter intervals between changes and the use of fine-pored or silicone-coated sponges.

In conclusion, this study demonstrates that ENPT is a valuable and effective salvage option for the treatment of AL after colorectal surgery, in patients for whom fecal diversion is not feasible and no further surgical therapy can be offered. The majority of patients can achieve successful outcomes within a few weeks, often avoiding the need for a permanent ostomy. A structured treatment approach, combined with a dedicated endoscopic team, is essential to optimize patient outcomes. Further research is warranted to further refine ENPT protocols and explore innovative approaches to treatment.

## Materials and methods

In this proof-of-concept study, all patients who had undergone colorectal surgery at the University Medical Center Hamburg-Eppendorf between June 2016 and February 2022 were screened. The differential diagnosis of AL was taken into consideration based on the clinical status (infectious signs, such as fever or sepsis manifestations) and laboratory findings (which represented an increase of the infection parameters, including leukocytosis or c-reactive protein). Further, the diagnosis of AL was confirmed using contrast enhanced computed tomography and endoscopic examination. Only patients who had endoscopically and/or radiologically confirmed AL with pelvic abscess (grade B according to the International Study Group of Rectal Cancer) were included^23^. Patients were excluded if they had undergone any other types of treatment, such as primary relaparotomy due to poor clinical conditions, CT-guided abscess drainage, rectal stenting, application of sealants (used for leakages < 2 mm via TISSEEL Fibrin Sealant^®^, Baxter™), and only irrigation of the leakage cavity without ENPT. All clinical data were obtained from a prospectively maintained database including clinical and endoscopic records and from records of follow-up visits by the patients to their physicians. The data obtained included leakage closure, recurrence of pelvic abscess and sepsis, rate of ostomy closure, and treatment-related complications. AL diagnosed during the first 4 weeks after operation were considered acute, while AL detected after 4 weeks was classified as chronic. The study was approved by the Ethics Committee of the Medical Chamber Hamburg and was performed in accordance with the ethical standards laid down in the 1964 Helsinki Declaration and its later amendments. The current study is in compliance with the STROCSS 2021 (strengthening of the reporting of cohort, cross-sectional and case-control studies in surgery)^24^.

### Diagnosis of anastomotic leakage

In cases of suspected AL, endoscopic examination and/or computed tomography (CT) was carried out. If leakage and/or pelvic abscess was confirmed and the patient was in stable condition, with no sign of peritonitis or need for surgical revision, ENPT was indicated. During the first endoscopic treatment session, rinsing and mechanical cleaning were performed and factors such as size of insufficiency (anastomotic defect), size of the abscess cavity, bowel vitality, visible vessels, and the presence of bowel inside the abscess cavity were documented. Endoscopic examinations were carried out using a standard gastroscope, except for patients with very small defects, in which case a 5.6-mm fine-caliber transnasal gastroscope (GIF XP 290 N, Olympus) was employed.

### Endoscopic negative pressure therapy

After endoscopic exploration of the leakage and the abscess cavity, in all cases an extraluminal intracavitary sponge was inserted into the abscess cavity via a tube, using forceps and a pulling-thread, or via a guide wire (Endo-Sponge^®^, B. Braun Melsungen, Germany). Depending on the size of the leak, the size of the abscess, and the degree of contamination, different materials were used for the application of the ENPT. Primary treatment began with the application of black sponge (3 M™ V.A.C.^®^ Granufoam ™, 3 M St. Paul, MN), which offers satisfactory wound cleaning and granulation inside the pelvic cavity thanks to its relatively large pores. However, the sponge needed to be changed after 2–5 days to prevent early ingrowth. In addition to black sponge, white sponge (3 M™ V.A.C.^®^ Granufoam™, 3 M St. Paul, MN), which is less adhesive, was used in patients with confirmed intraperitoneal leakage to avoid bleeding complications or intestinal. An illustrative case is depicted in Supplementary Fig. 1.

### Open-pore film drainage

Open-pore film (OPF) drainage (Suprasorb CNP, Lohmann & Rauscher, Germany), first described by Loske, was indicated in patients with small-diameter, fistula-like defects^25,26^. This technique can be used when there is no longer enough space to place a sponge via the endoscope, due to reduction in the size of the abscess cavity in the later stages of healing. Individually fashioned catheters with OPF drainage could be left in place for a longer time. This technique represents a further development of negative-pressure treatment, transferring its application from external use on the body surface to internal use with the aid of flexible endoscopy. Endoscopic vacuum therapy (EVT) is initially introduced for the treatment of rectal anastomotic insufficiency and was later applied in the postoperative management of complications in the upper gastrointestinal tract. Through precise endoscopic evaluation, it is possible to create fast, simple, and individually optimized drainage systems for insertion into abscess cavities, fistula tracts, or intestinal defects. In most cases, EVT tubes will be replaced at each treatment session, reflecting the progressive improvement of the local condition. For large, contaminated abscess cavities, one or more black sponges will be used initially. For narrow, long fistula-like tracts, Suprasorb-CNP film drains will be employed. We changed them once a week in most patients. An example is shown in Supplementary Fig. 2.

### Negative pressure application and modification of the suction material

The management of AL in two individual patients is described in detail in the supplementary materials. In all cases we used an electronic pump (3 M, St. Paul, MN) with a pressure of − 125 mmHg to set up the suction. Black sponges were changed endoscopically twice each week, while in the case of white sponge or OPF drainage we left the tube in place for up to 8 days. Depending on the amount of necrotic tissue, rinsing of the cavity and mechanical debridement were carried out with a forceps or a brush. If an ostomy is not present and fecal passage is preserved, the sponge within the abscess cavity can quickly become obstructed. Therefore, the intestinal lumen was irrigated per rectum several times a day as needed during treatment, using enemas, rectal irrigators, decompression tubes, or irrigation systems. If these measures were insufficient, an “anti-fecal” shield was employed. In this approach, an OPF and a black sponge were combined to create a shield for the lumen. The black sponge covers the orifice, adhering effectively due to its strong suction properties, and prevents fecal contamination of the underlying fistula or abscess. This method typically leads to the collapse of the abscess cavity and the formation of a long, thin fistula, which can be progressively reduced until complete closure is achieved.

The treatment continued until the patient’s clinical condition was stable and the abscess cavity was clean with granulation tissue. In the case of a persisting wound cavity, transanal rinsing was conducted as described earlier^20^. Beside the ENPT with black or white sponge or OPF, additional endoscopic methods were used in individual selected cases: intestinal rinsing (decompression tubes, irrigation systems, etc.), endoscopic AL closure after ENPT by direct suture, tulip bundle maneuver with a loop and clips, or Vicryl mesh.

### Statistical analysis

Statistical analyses were carried out using IBM^©^ SPSS^©^ Statistics for Mac (Version 20, IBM Corporation, Armonk, NY, USA). Patient characteristics were expressed in terms of median with interquartile range or mean with minimum and maximum. After exploration, statistical evaluation of continuous data was performed using the non-parametric Mann–Whitney test. For categorical data, the χ^2^ test and Fisher’s exact test were used. All tests were two-sided, and the level of statistical significance was set at *p* < 0.05.

## Supplementary Information

Below is the link to the electronic supplementary material.

Supplementary Material 1

## Data Availability

The datasets generated and/or analyzed during the current study are available from the corresponding author upon reasonable request.

## Declarations

### Approval for human experiments

Informed consent was obtained from all subjects and/or their legal guardian(s).

## References

[1] Karliczek, A. et al. Drainage or nondrainage in elective colorectal anastomosis: a systematic review and meta-analysis. *Colorectal Dis.* **8**, 259–265. 10.1111/j.1463-1318.2006.00999.x (2006). 16630227 10.1111/j.1463-1318.2006.00999.x

[2] Kessler, H., Hermanek, P. Jr. & Wiebelt, H. Operative mortality in carcinoma of the rectum. Results of the German multicentre study. *Int. J. Colorectal Dis.* **8**, 158–166. 10.1007/bf00341191 (1993). 8245673 10.1007/BF00341191

[3] Cong, Z. J. et al. Influencing factors of symptomatic anastomotic leakage after anterior resection of the rectum for cancer. *World J. Surg.* **33**, 1292–1297. 10.1007/s00268-009-0008-4 (2009). 19363687 10.1007/s00268-009-0008-4

[4] Ju, H. E. et al. High incidence of late anastomosis leakage in patients for rectal cancer after neoadjuvant chemoradiotherapy: A comparative study. *Asian J. Surg.* **45**, 1832–1842. 10.1016/j.asjsur.2021.10.039 (2022). 34815142 10.1016/j.asjsur.2021.10.039

[5] Daams, F., Wu, Z., Lahaye, M. J., Jeekel, J. & Lange, J. F. Prediction and diagnosis of colorectal anastomotic leakage: A systematic review of literature. *World J. Gastrointest. Surg.* **6**, 14–26. 10.4240/wjgs.v6.i2.14 (2014). 24600507 10.4240/wjgs.v6.i2.14 PMC3942535

[6] Doeksen, A. et al. Radiological evaluation of colorectal anastomoses. *Int. J. Colorectal Dis.* **23**, 863–868. 10.1007/s00384-008-0487-z (2008). 18560844 10.1007/s00384-008-0487-z PMC2493516

[7] Ortega-Deballon, P. et al. C-reactive protein is an early predictor of septic complications after elective colorectal surgery. *World J. Surg.* **34**, 808–814. 10.1007/s00268-009-0367-x (2010). 20049435 10.1007/s00268-009-0367-x PMC2877195

[8] Doerfer, J. et al. The importance of radiological controls of anastomoses after upper Gastrointestinal tract surgery - a retrospective cohort study. *Patient Saf. Surg.* **4**10.1186/1754-9493-4-17 (2010). 10.1186/1754-9493-4-17 PMC2994795 21070633

[9] Arezzo, A. et al. Long-term efficacy of endoscopic vacuum therapy for the treatment of colorectal anastomotic leaks. *Dig. Liver Dis.* **47**, 342–345. 10.1016/j.dld.2014.12.003 (2015). 25563812 10.1016/j.dld.2014.12.003

[10] Jagielski, M., Piątkowski, J., Jarczyk, G. & Jackowski, M. Transrectal endoscopic drainage with vacuum-assisted therapy in patients with anastomotic leaks following rectal cancer resection. *Surg. Endosc*. **36**, 959–967. 10.1007/s00464-021-08359-4 (2022). 33650007 10.1007/s00464-021-08359-4 PMC8758650

[11] Kühn, F. et al. Endoscopic vacuum therapy for the treatment of colorectal leaks - a systematic review and meta-analysis. *Int. J. Colorectal Dis.* **37**, 283–292. 10.1007/s00384-021-04066-7 (2022). 34817647 10.1007/s00384-021-04066-7 PMC8803669

[12] Strangio, G. et al. Endo-sponge therapy for management of anastomotic leakages after colorectal surgery: A case series and review of literature. *Dig. Liver Dis.* **47**, 465–469. 10.1016/j.dld.2015.02.007 (2015). 25769505 10.1016/j.dld.2015.02.007

[13] Sharp, G., Steffens, D. & Koh, C. E. Evidence of negative pressure therapy for anastomotic leak: a systematic review. *ANZ J. Surg.* **91**, 537–545. 10.1111/ans.16581 (2021). 33480168 10.1111/ans.16581

[14] Şandra-Petrescu, F., Tzatzarakis, E., Kähler, G., Reissfelder, C. & Herrle, F. Management of colorectal anastomotic leakage using endoscopic negative pressure therapy with or without protective ostomy: a retrospective study. *Int. J. Colorectal Dis.* **36**, 2261–2269. 10.1007/s00384-021-04011-8 (2021). 34455472 10.1007/s00384-021-04011-8 PMC8426235

[15] Schiffmann, L. et al. Neoadjuvant radio-chemotherapy prolongs healing of anastomotic leakage after rectal resection treated with endoscopic vacuum therapy. *Th. Adv. Gastroenterol.* **12**, 1756284819877606. 10.1177/1756284819877606 (2019). 10.1177/1756284819877606 PMC6759710 31579099

[16] Baum, P. et al. Mortality and complications following visceral surgery: A nationwide analysis based on the diagnostic categories used in German hospital invoicing data. *Dtsch. Arztebl Int.* **116**, 739–746. 10.3238/arztebl.2019.0739 (2019). 31774053 10.3238/arztebl.2019.0739 PMC6912125

[17] Wu, S. W., Ma, C. C. & Yang, Y. Role of protective stoma in low anterior resection for rectal cancer: a meta-analysis. *World J. Gastroenterol.* **20**, 18031–18037. 10.3748/wjg.v20.i47.18031 (2014). 25548503 10.3748/wjg.v20.i47.18031 PMC4273155

[18] Vignali, A. & De Nardi, P. Endoluminal vacuum-assisted therapy to treat rectal anastomotic leakage: A critical analysis. *World J. Gastroenterol.* **28**, 1394–1404. 10.3748/wjg.v28.i14.1394 (2022). 35582677 10.3748/wjg.v28.i14.1394 PMC9048477

[19] Lange, J. et al. The vacstent trial: combined treatment of esophageal leaks by covered stent and endoscopic vacuum therapy. *Surg. Endosc*. **37**, 3657–3668. 10.1007/s00464-023-09861-7 (2023). 36639580 10.1007/s00464-023-09861-7 PMC10156910

[20] Kantowski, M. et al. Improved colorectal anastomotic leakage healing by Transanal rinsing treatment after endoscopic vacuum therapy using a novel patient-applied rinsing catheter. *Int. J. Colorectal Dis.* **35**, 109–117. 10.1007/s00384-019-03456-2 (2020). 31792582 10.1007/s00384-019-03456-2

[21] Grund, K. E., Schweizer, U., Zipfel, A. & Duckworth-Mothes, B. Learning of flexible endoscopy, particularly endoscopic vacuum therapy (EVT). *Chirurg* **93**, 56–63 10.1007/s00104-021-01497-4 (2022). 10.1007/s00104-021-01497-4 34570261

[22] Mahendran, B., Rossi, B., Coleman, M. & Smolarek, S. The use of Endo-SPONGE(^®^) in rectal anastomotic leaks: a systematic review. *Tech. Coloproctol*. **24**, 685–694. 10.1007/s10151-020-02200-1 (2020). 32377984 10.1007/s10151-020-02200-1

[23] Rahbari, N. N. et al. Definition and grading of anastomotic leakage following anterior resection of the rectum: a proposal by the international study group of rectal cancer. *Surgery* **147**, 339–351. 10.1016/j.surg.2009.10.012 (2010). 20004450 10.1016/j.surg.2009.10.012

[24] Mathew, G. et al. STROCSS 2021: strengthening the reporting of cohort, cross-sectional and case-control studies in surgery. *Int. J. Surg.* **96**, 106165. 10.1016/j.ijsu.2021.106165 (2021). 34774726 10.1016/j.ijsu.2021.106165

[25] Loske, G. et al. Successful endoscopic vacuum therapy with new open-pore film drainage in a case of iatrogenic duodenal perforation during ERCP. *Endoscopy* **47**, E577–e578. 10.1055/s-0034-1393388 (2015). 26649468 10.1055/s-0034-1393388

[26] Loske, G. et al. Open-pore film drainage (OFD): a new multipurpose tool for endoscopic negative pressure therapy (ENPT). *Endosc Int. Open.* **6**, E865–e871. 10.1055/a-0599-5886 (2018). 29978007 10.1055/a-0599-5886 PMC6031437

