# Supplementary material for: Endoscopic negative pressure therapy as a salvage treatment for management of post-surgical anastomotic leaks without ostomy after colorectal resection
Source: Sci Rep. 2025 Oct 31;15:38115. doi: 10.1038/s41598-025-25181-3 (PMC12579235; doi:10.1038/s41598-025-25181-3)
Supplement: Supplementary file 1 — Supplementary Material 1 [file 41598_2025_25181_MOESM1_ESM.docx]

**Endoscopic Negative Pressure Therapy as a Salvage Treatment for Management of Post-Surgical Anastomotic Leaks Without Ostomy after Colorectal Resection**

**(Supplementary material)**

Kantowski M^1, 3^, Perez D^2^, Bellon E^2^, Rösch T^1^, Ramouz A^3^, Tachezy M^2^^*^, Scognamiglio P ^2*^

^1^ Department of Interdisciplinary Endoscopy, University Medical Center Hamburg-Eppendorf, 20248 Hamburg, Germany

^2^ Department of General, Visceral and Thoracic Surgery, University Medical Center Hamburg-Eppendorf, 20248 Hamburg, Germany

^3^ Department of Surgery, Heidelberg University Hospital, 69120 Heidelberg, Germany

*Joint last authors

Kantowski M: ORCID-ID: 0009-0000-3694-9005

Conflict of Interest: None

Corresponding author:

Marcus Kantowski, MD

Department of General, Visceral and Thoracic Surgery

University-Hospital of Heidelberg, Germany

Im Neuenheimer Feld 420

69120 Heidelberg, Germany

Phone: +49 6221-566110

Email: [marcus.kantowski@med.uni-heidelberg.de](mailto:marcus.kantowski@med.uni-heidelberg.de)

**Supplementary figure 1:**

**Example of percutaneous endoscopic access and ENPT**


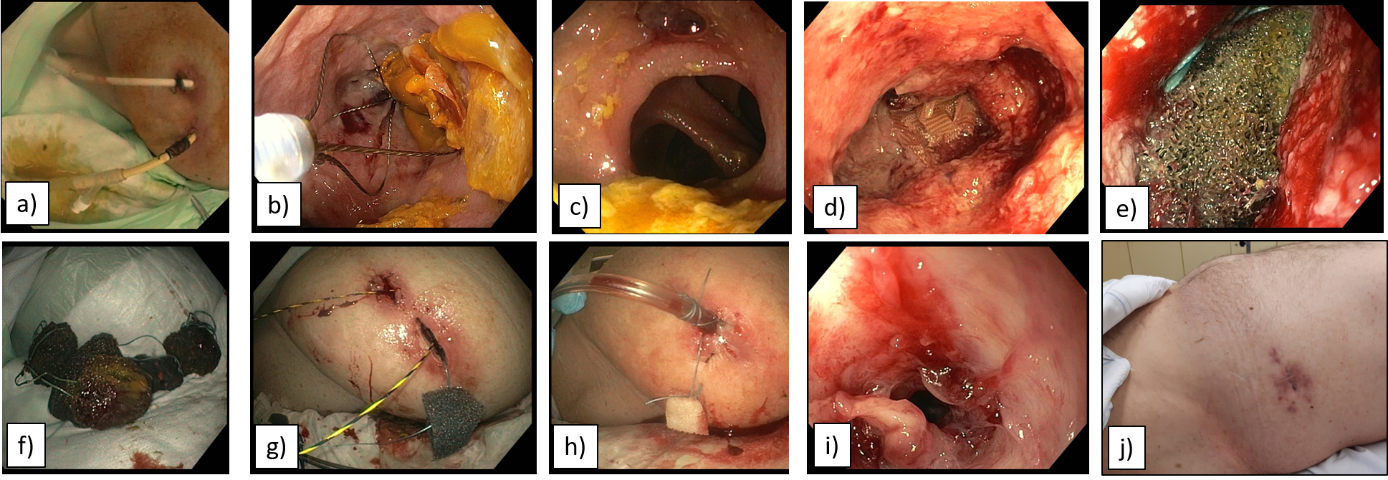


Patient with sigmoid leakage after severe diverticulitis and covered perforation with multiple adhesions. Reoperation to perform a new sigmoid anastomosis ended unsuccessfully with a residual small AL and a localized abdominal abscess. Sufficient closing of the endoluminal sigma anastomotic leakage with an over-the-scope clip. (a) CT-guided abscess drainage with two drains. Some days later feculent drainage secretion occurred. High-risk reoperation should be avoided in this patient. (b) Both drainage tubes were removed under radiological guidance. Stable percutaneous access was opened to insert a standard gastroscope up to 10 mm in diameter into the hand-sized abscess in the left lower abdomen. Food and stool were removed mechanically via the gastroscope. (c) An intestinal defect 12 mm in diameter caused by the abdominal abscess was found. (d) A resorbable Vicryl net (Ethicon, Norderstedt) was endoscopically placed inside the abscess to close the intestinal defect; the net was replaced twice each week. (e) The abscess cavity was cleaned by ENPT using a “pearl necklace” of black sponge on a thread to achieve rapid reduction of abscess size. (f) Black sponge after removal. (g) A guide wire is placed inside the abscess cavity for the placement of the suction tube covered with OPF on the distal tip, and the last black sponge is placed outside the body fixed on a thread for easy removal twice each week and insertion of the next “pearl necklace”. (h) After initial cleaning of the contaminated abscess cavity, the black sponge are exchanged for white sponge with lower risk of further intestinal erosion. (i) Shrinking of the abscess to a small tunnel. (j) Result after 3 weeks of percutaneous ENPT without a third reoperation or ostomy.

**Supplementary figure 2:**

**Healing process by percutaneous ENPT using black/white sponge and OPF drainage**


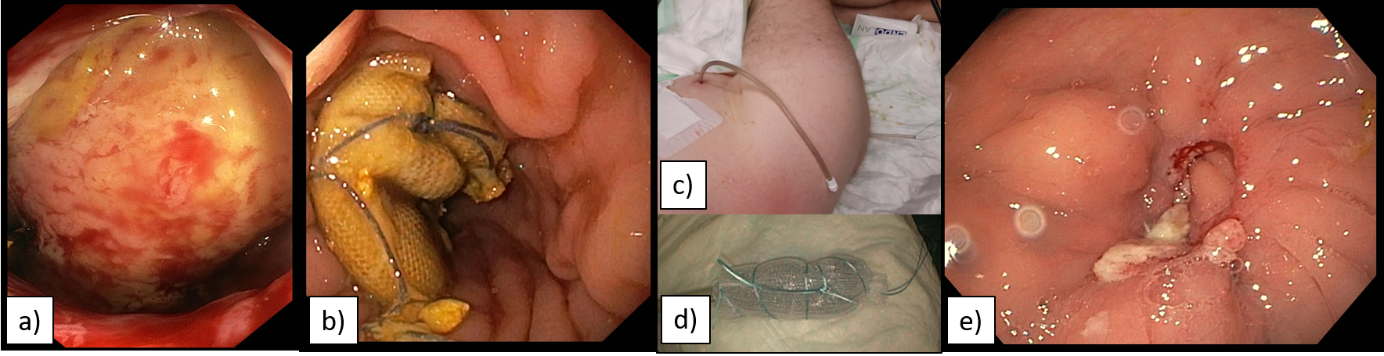


On the left side a colorectal anastomosis (pink) with an AL is shown. Percutaneous access is chosen, using the old channel from a drainage tube placed during the index operation. Of course, the ENPT tube could be removed via the rectum. First, the hole (black) is sealed with an endoscopically placed resorbable Vicryl net, which is changed twice each week (yellow). Behind the leakage a pelvic abscess cavity is found (green) with an old drainage channel to the skin (white). (a) At the start of the treatment the abscess cavity is filled with several black sponges, all fixed on a thread. The last sponge of the thread is placed outside the body for easy complete removal. Suction drainage of the abscess is established by a small tube covered with OPF on the distal tip for optimized suction to avoid early tube occlusion by fibrin. (b) The AL defect is getting smaller. The size of the Vicryl net could be reduced in the course of time, as could the number and size of the black sponges. (c and d) Because large vessels are directly visible on other intestinal organs inside the abscess cavity, the black sponges are exchanged for white sponges to avoid long-term complications of suction treatment such as severe bleeding or a second intestinal fistula. (e) After complete closure of the AL defect a Vicryl net is no longer needed. The initial large abscess cavity has been transformed into a small channel. To avoid a secondary late abscess in this channel the suction tube is left in place for a further few days before eventual removal.
